# Nucleotide Salvage Deficiencies, DNA Damage and Neurodegeneration

**DOI:** 10.3390/ijms16059431

**Published:** 2015-04-27

**Authors:** Michael Fasullo, Lauren Endres

**Affiliations:** Colleges of Nanoscale Sciences and Engineering, State University of New York Polytechnic University, Albany, NY 12203, USA; E-Mail: Lendres@sunycnse.com

**Keywords:** nucleotide salvage, neurodegenerative diseases, mitochondrial DNA stability, genetic stability

## Abstract

Nucleotide balance is critically important not only in replicating cells but also in quiescent cells. This is especially true in the nervous system, where there is a high demand for adenosine triphosphate (ATP) produced from mitochondria. Mitochondria are particularly prone to oxidative stress-associated DNA damage because nucleotide imbalance can lead to mitochondrial depletion due to low replication fidelity. Failure to maintain nucleotide balance due to genetic defects can result in infantile death; however there is great variability in clinical presentation for particular diseases. This review compares genetic diseases that result from defects in specific nucleotide salvage enzymes and a signaling kinase that activates nucleotide salvage after DNA damage exposure. These diseases include Lesch-Nyhan syndrome, mitochondrial depletion syndromes, and ataxia telangiectasia. Although treatment options are available to palliate symptoms of these diseases, there is no cure. The conclusions drawn from this review include the critical role of guanine nucleotides in preventing neurodegeneration, the limitations of animals as disease models, and the need to further understand nucleotide imbalances in treatment regimens. Such knowledge will hopefully guide future studies into clinical therapies for genetic diseases.

## 1. Introduction

Maintaining the appropriate level and balance of nucleotides is critical for DNA integrity and preventing neurodegeneration. Nucleotide levels are maintained in eukaryotes by *de novo* synthesis and nucleotide salvage; the latter mechanism is particularly important in neural cells (for review, see [1]). DNA damage triggers an increase in deoxynucleoside triphosphate (dNTP) levels, which is necessary for DNA repair mechanisms that require unscheduled DNA synthesis [2]. In model organisms, such as budding yeast, inability to increase dNTP levels after DNA damage exposure leads to higher frequencies of genetic instability [2]. Yeast cells that cannot maintain dNTP levels exhibit higher frequencies of spontaneous and DNA damage-associated petite colonies, due to mitochondrial dysfunction [3,4]. The mitochondrial genome is especially prone to damage due to incorporation of 8-oxo-deoxyguanosine triphosphate (8-oxo-dGTP), a mutagenic nucleotide resulting from oxidative stress. Similarly, insufficient dNTP levels result in mitochondrial and chromosomal instability in higher eukaryotic cells [5]. The purpose of this review is to correlate deficiencies in nucleotide salvage and synthesis with neurological and DNA metabolism defects.

Mutations in single genes encoding defective metabolic enzymes have been associated with neurological pathologies and purine nucleotide salvage, resulting in chronic gout, neurodegeneration, and odd behavioral pathologies, including self-mutilation by biting (for review, see [6]). Mitochondrial DNA depletion syndrome (MDS) has been associated with nine nuclear genes involved in the maintenance of mitochondrial dNTP pools, including thymidine kinase 2 (TK2), deoxyguanosine kinase (DGOUK), p53 dependent ribonucleotide reductase subunit 2 (RRM2B) and thymidine phosphorylase (TYMP); the most severe forms of the disease lead to infantile death (for review, see [7,8]). Mitochondrial neurogastrointestinal encephalomyopathy (MNGIE) is a rare autosomal disease that is associated with a defect in TYMP, but is late in onset; mitochondrial mutations are associated with ophthalmoparesis aberrant behavior. For example, Lesch-Nyhan syndrome (LNS) is a devastating, rare X-linked disease, in which defective, gastrointestinal dysmotility, peripheral neuropathy, and leukoencephalopathy occurs [9]. Although the deficient repair of DNA double-strand breaks and X-ray sensitivity are primarily associated with defects in ataxia telangiectasia mutated (ATM), ataxia telangiectasia (A-T) patients are defective in deoxycytosine salvage [10], and exhibit defective mitochondria [11], and cerebellar degeneration [12,13]. Underlying questions for each of these diseases concern the variation in severity of clinical pathologies and timing of onset, how mutations in the same gene can lead to different clinical presentations, and whether restoring dNTP levels or balance results in cure (Table 1). To understand these pathologies, it is worthwhile to review the role of dNTPs in neurological physiology, the basic biochemistry by which purine and dNTPs are salvaged and maintained, cellular defects that result when dNTPs are imbalanced, and mouse disease models. Elucidating the molecular defects can then guide strategies for gene therapy and possible drug treatments. This review explores current knowledge of how defects in nucleotide metabolism may associate with DNA damage and repair mechanisms in neurological tissue. In this review, we will discuss the historical background of nucleotide salvage and neurological disease, the biochemical control of pathways involved in nucleotide salvage and in maintaining dNTP levels, the checkpoint control of nucleotide salvage, insights from model organisms, recent mouse studies, and finally, discrepancies in the mouse and human models and future directions.

**Table 1:** Nucleotide metabolism genes and associated genetic defects.

Gene	Disease	Frequency	Mode of Inheritance	Enzymatic Defect	Neurophathy in Selected Patients	Effect on Mitochondria	Pathologies/Onset
*HPRT1*	Lesch-Nyhan	Rare, >300 alleles 1/380,000 live births	X-linked	Hypoxanthine guanine phosphoribosyl transferase	Motor function	Indirect production of free radicals	Gout, diminished IQ, dystonia, Death due to hypotonia or renal failure
*DGUOK*	Mitochondrial depletion syndrome (MDS); hepatocerebral form	Extremely Rare, 22 different mutations described	Autosomal recessive	Deoxy-guanosine kinase	Hearing loss, nystagmus	Mitochondria depletion due to failure to produce substantial dGTP	Progressive liver failure, patients generally die of liver failure in early childhood
*TK2*	MDS (myopathic form)	Rare	Autosomal recessive	Thymidine kinase II	Hypotonia, neurological features	Mitochondria depletion due to failure to salvage thymidine in mitochondria	Muscle weakness, extreme forms lead to respiratory failure and infantile death; mutations in less conserved aminoacids lead to progressive external ophthalmoplegia
	Progressive external ophthalmoplegia (PEO)						
*TYMP* (*ECGF1*)	Mitochondrial neurogastroIntestinal encephalomyopathy (MNGIE)	Rare, 50 different mutations have been described	Some domininant alleles	Thymidine phosphorylase	Peripheral neuropathy, ophthalmoparesis, leukoencephalopathy	Mitochondrial depletion	Gastrointestinal dysmotility, weight loss; onset within first 20 years, 37 years the median age of death
*RRM2B*	MDS (Encephalomyopathic form)	Rare	Recessive alleles lead to severe MDS or MNGIE; dominant truncations confer PEO	p53-induced ribonucleotide reductase B subunit (p53R2)	Microcephaly,sensorineural hearing loss, ophthalmoplegia	Mitochondria depletion; failure to repair UV damage	Severe skeletal depletion of mitochondria, hypotonia and muscle weakness, sensorineural hearing loss, failure to thrive, lactic acidosis; mutations that confer PEO or MGIE are adult onset
	PEO						
	MNGIE						
*ATM*	Ataxia Telangiectasia (A-T)	Rare	Autosomal recessive	ATM	Cerebellar ataxia	Mitochondria less-well formed	X-ray sensitivities, High frequencies of lymphoma/pulmonary infection

See references [7,8,9,10,11] for detailed discussions.

Nucleotide metabolism must be finely regulated in the nervous system, since although differentiated neuronal cells are non-cycling, there is high level of metabolism and large demand for ATP [14]. Whereas astrocytes are glycolytic and can produce lactate, neurons derive most of their energy through oxidative phosphorylation and can consume the lactate produced by astrocytes [15]. Thus, there is a demand for high mitochondrial copy number that depends on the replication and integrity of the 16.5 kb mitochondrial genome. However, cellular respiration catalyzed by oxidative phosphorylation can be an internal source of reactive oxygen species (ROS), which include superoxide anions, hydroxyl radicals, and hydrogen peroxide [16]. While ROS can directly damage DNA, they can also generate oxidized dNTPs, such as 8-oxo-dGTP, which can then be incorporated into either mitochondrial or genomic DNA, creating genetic instability [16,17]. Replication fidelity of polymerase γ, the mitochondrial DNA polymerase, is greatly affected by trace amounts of 8-oxo-dGTP [18]. Because mitochondria are involved in Ca^2+^ homeostasis, fatty acid metabolism, and pyrimidine nucleotide synthesis, mitochondrial dysfunction can lead to devastating pleiotropic effects and trigger apoptosis [19]. To maintain and balance dNTP levels, cells contain a specialized form of ribonucleotide reductase for *de novo* synthesis of dNTPs, encoded by RRM2B [20,21], and two specific salvage enzymes, thymidine kinase II (TK2) and deoxyguanosine kinase (dGK), which are located in the mitochondria.

Besides serving as energy co-factors and building blocks for RNA and DNA, nucleotides and bases also have critical roles in cell physiology as signaling molecules [22]. cAMP critically modulates the development of neuronal connectivity [23]. ATP is an important co-stimulator molecule in motor, sensory-motor, hypothalamus, parasympathetic and sympathetic nerves, functions in neuroprotection, and participates in neuro-regeneration from stem cells [23]. Guanosine modulates glutamatergic neurotransmission by stimulating glial reuptake of l-glutamate [24]. These examples illustrate that nucleotides can also serve as signaling molecules in order to facilitate neural synapses.

## 2. Biochemistry of *de Novo* and Salvage Pathways for Nucleotide Biosynthesis in the Nervous System

**Figure 1:**
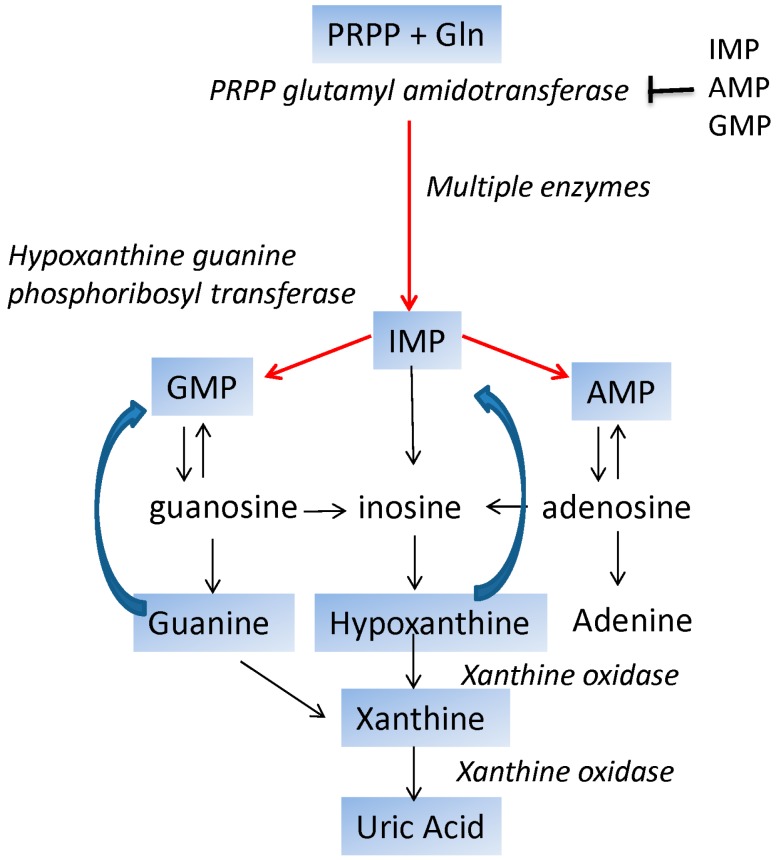
Outline of purine *de novo* synthesis, salvage, and degradation. Red arrows indicate sysnthesis, and blue arrows indicate salvage. Black arrows indicate the conversion of nucleotides to nucleosides and bases, and the conversion of nucleosides to nucleotides. Phosphoribosyl pyrophosphate (PRPP) is inhibited by adenylate (AMP), inosylate (IMP), and guanylate (GMP), as indicated.

Because there is a dynamic pool of dNTPs, dNTPs must be continually synthesized and degraded. dNTPs can also be recycled from free bases resulting from RNA or DNA turnover. Since terminally differentiated neurons do not replicate, in order to maintain nucleotide levels and balance there is greater reliance on salvage pathways compared to *de novo* synthesis [6]. In *de novo* synthesis, purine and pyrimidine ribonucleotides are made from basic constituents; these include phosphoribosyl pyrophosphate (PRPP), glutamine, and glycine for purine nucleotides, and carbamoyl phosphate, aspartate, and PRPP for pyrimidine biosynthesis. The key regulated enzyme in *de novo* purine biosynthesis is glutamine PRPP amidotransferase, which is feedback inhibited by the ribonucleotide monophosphates, adenylate (AMP), inosylate (IMP), and guanylate (GMP), and positively activated by PRPP (Figure 1).

Pyrimidine *de novo* biosynthesis is generally regulated at the step of carbamoyl phosphate synthetase, and key initial enzymatic reactions are encoded in one large cytoplasmic polypeptide, composed of carbomoyl phosphate synthetase, aspartate transcarbamoylase, and dihyrooratase (CAD, for general information concerning nucleotide biosynthesis, see [25]).

Ribonucleoside diphosphates (NDPs) are made and then converted to deoxynucleoside diphosphates (dNDPs) by reduction catalyzed by the allosteric enzyme ribonucleotide reductase (RNR, [21]). RNR is a tetramer composed of two small subunits, which contain the catalytic site, and two large subunits, which contain the substrate binding sites for NDPs, as well as regulatory binding sites for deoxyadenosine triphosphate (dATP), deoxythymidine triphosphate (dTTP), and deoxyguanosine triphosphate (dGTP) [21]. While dATP allosterically acts to inhibit the activity of RNR, dTTP allosterically activates the conversion of purine ribonucleotides and allosterically inhibits the conversion of cytosine diphosphate CDP. RNR subunits are regulated at the level of transcription, translation, and post-translational modification to ensure that essential dNTPs meet the demand of DNA synthesis [21].

In neural tissue, salvage pathways are important to maintain the nucleotide balance, and the utilization of *de novo* pathways diminish towards adulthood. The free purine bases, hypoxanthine and guanine, can be salvaged by hypoxanthine guanine phosphoribosyl transferase (HGPRT). The products of HGPRT include the PRPP amidotransferase allosteric inhibitors IMP, GMP, AMP. The reaction also utilizes the substrate PRPP, the allosteric positive activator or PRPP amidotransferase. Thus, HGPRT salvages bases while at the same time reducing *de novo* synthesis of purine nucleotides by increasing the concentration of inhibitory nucleotides and decreasing the concentration of PRPP. HGRPT phosphoribosyl transferase is located in the nervous system and is found in abundance in the brain [26].

**Figure 2:**
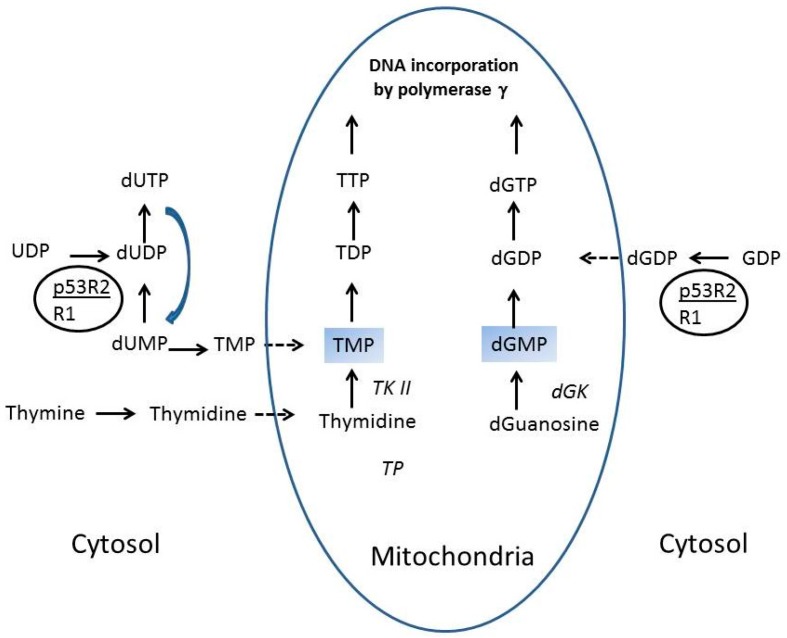
Key steps in the salvage of deoxynucleotides in the mitochondria. Arrows designate the steps in the salvage pathways; a dashed arrow indicates import into the mitochondria, and a blue arrow indicates degradation. The large oval (blue) is the mitochondria, the small oval (black) represents the p53-regulated ribonucleotide reductase (p53R2/R1). The nucleotides in the mitochondria include thymidylate (TMP), thymidine diphosphate (TDP), thymdine triphosphate (TTP), deoxyguanosine monophosphate (dGMP), deoxyguanosine diphosphate (dGDP), and deoxyguanosine triphosphate (dGTP). Thymidine kinase II (TK2) and deoxyguanosine kinase (dGK) are salvage enzymes.

Salvage pathways for deoxynucleotides do not involve phosphoribosyl transferases, but instead involve kinases that convert nucleosides to nucleotides (Figure 2). The thymine salvage pathway generating thymidylate involves nucleoside phosphorylase, which can catalyze either the production of the nucleoside or of the base and sugar, which could then be degraded. However, in the presence of thymidine kinase, thymidylate (TMP) synthesis shifts the catalysis towards thymine salvage. Similarly, guanosine kinase and cytidine kinase can generate deoxyguanylate (dGMP) and deoxycytidylate (dCMP), respectively [27].

The degradation of nucleotides is important in maintaining nucleotide pools [27]. Generally pyrimidine nucleotides are degraded by nucleotidases and nucleoside phosphorylases, which cleave the base from the sugar; the pyrimidine bases can be completely degraded to CO_2_ and H_2_O. However, purine bases cannot be completely degraded and the ultimate end product is uric acid (Figure 1). Guanine nucleotides are degraded to hypoxanthine and xanthine, respectively. Hypoxanthine and xanthine are substrates of xanthine oxidase, generating hydrogen peroxide and uric acid. Thus, overactive xanthine oxidase leads to excess uric acid and the production of ROS [28]. Uric acid has low solubility in the blood, and uric acid crystals can trigger gout resulting from extreme inflammation [29] (Figure 1).

Deoxynucleotide pools in the mitochondria are maintained by both cytoplasmic *de novo* enzymes and salvage enzymes located with the mitochondria (Figure 2). In general, mitochondrial deoxynucleotide levels are approximately ten-fold lower than levels measured from the cytoplasm [30,31,32,33]. A cytoplasmic ribonucleotide reductase which is not found in cycling cells is found in neuronal tissue in which the small subunit R2 is substituted by the p53R2 [20].

Nucleotide transporters then shuttle nucleotides into the mitochondria. In addition, the mitochondrial forms of guanosine kinase and thymidine kinase, although encoded by nuclear genes, are active in the mitochondria. Interestingly, mRNA expression of deoxyguanosine kinase is highest in muscle, brain, liver, and lymphoid tissues, where there is high demand for mitochondrial function. Measurements of mitochondrial dNTP concentrations in human fibroblasts and liver indicate that dGTP is the most abundant dNTP [30,31,32,33], and that much of the dGTP is derived, however, from the *de novo* synthesis catalyzed in the cytoplasm [34]. These studies have indicated that both ribonucleotide reductase and salvage enzymes contribute to maintaining deoxynucleotide levels in the mitochondria.

## 3. Human Diseases Resulting from Defective Nucleotide Metabolism

### 3.1. Lesch-Nyhan Disease

Lesch-Nyhan syndrome (LNS), first reported in 1964 [6], affects 1/380,000 live births and is due to mutations in the X-linked *HPRT1* gene, resulting in defective hypoxanthine guanine phosphoribosyl (HPRT) transferase activity [6,34]. Over three hundred alleles have been described, some of which result from large deletions due to recombination between Alu repeats [34,35]. The disease has three major characteristics: (1) overproduction of uric acid; (2) motor and cognitive disability; and (3) self-injuring behavior. The neurological and behavioral symptoms of the disease are less severe in some cases, and molecular genetics suggest that this may depend on the residual HPRT activity; however all forms are characterized by chronic over-production of uric acid. Extreme neurological defects include an inability to walk [36,37]. Patients have a shorter life span, and mortality is generally due to renal failure and hypotonia in the first or second decade of life; however, less severe cases may have longer lifespans [24,36].

The behavior and neurological symptoms have puzzled clinicians since the inherited disease is closely linked to a single metabolic defect in purine nucleotide salvage. Treatment with allopurinol that inhibits xanthine oxidase does reduce plasma uric acid levels but does not alleviate the neurological symptoms. A breakthrough was reported by observations that LNS patients have abnormally few dopaminergic nerve terminals [38]. The abnormality involves all dopaminergic pathways and is not restricted to the basal ganglia, as confirmed by positron emission tomography (PET) scans [39]. The developmental nature of the origin of the dopaminergic deficits suggests that these defects may explain both the behavioral and neurological symptoms of the disease [38].

There is no clear explanation for how the scarcity of dopaminergic nerve terminals results from the purine salvage defect. One hypothesis is based on the observations that because of the purine salvage deficiency, cells produce high levels of hypoxanthine but lower levels of GTP. Since GTP is the precursor of tetrahydrobiopterin (BH4) and conversion of tyrosine to dopamine requires BH4, dopamine synthesis may be insufficient [40,41]. In addition, high levels of hypoxanthine may be toxic to neurotransmission since hypoxanthine can bind to the benzodiazepine agonist recognition site on the γ-aminobutyric acid-A (GABA-A) receptor complex [40]. These data thus indicate that multiple mechanisms could adversely affect neurological function due to nucleotide imbalance.

Several studies suggest that LNS patients are defective in DNA repair [42,43]. ATP-ribose activity is diminished in fibroblasts derived from LNS, and would affect base excision DNA repair mechanisms, which are important for maintaining mitochondria. Additional experiments using HPRT-defective pluripotent ESD3 murine embryonic stem cells demonstrate that the transcriptional profile of the differentiating stem cells show down regulation of DNA repair and cell cycle genes, as well as a shift in the developmental profile from neuronal to a glial pattern [44]. These studies indicate that multiple signaling pathways that affect neuronal development may be altered in LNS patients and that the complex neurological and behavioral symptoms may be due to a combination of factors resulting from nucleotide imbalance [45,46,47,48].

### 3.2. Mitochondrial Depletion Syndromes (MDS)

The rare autosomal recessive mitochondrial deletion syndromes (MDS) include a range of genetic defects that share in common the depletion of mitochondrial DNA. Among this group are several diseases that affect deoxynucleotide salvage and *de novo* synthesis [48,49]. The three main clinical forms of MDS, myopathic, encephalomyopathic and hepatocerebral, are defined through the tissues that they affect. Mitochondrial neurogastrointestinal encephalomyopathy (MNGIE) affects the gastrointestinal tract [48,49]. Progressive external ophtalmoplegia (PEO) is the paralysis or weakness of the eye muscles [43,49,50]. Although many of these diseases may result in early childhood death, the clinical severity may correlate with the biochemical defect.

The hepatocerebral form of MDS resulting from mutations in the gene encoding deoxyguanosine kinase (dGUOK) [51], is a rare recessive autosomal disease with devastating clinical effect [50]. Twenty-two point mutations, deletions and insertions in dGUOK have been reported [52,53]. Friesinger *et al.* reported six patients with mutations in dGUOK [54]. Symptoms include dGUOK-related neonatal hepatic dysfunction, such as lactic acidosis and hypoglycemia, and neurological dysfunction, including psychomotor delay, hypotonia, and rotary nystagmus [50]. Individual patients have also been noted to experience progressive hearing loss. Most patients die in early childhood due to liver failure, although a few have been noted to survive into teen years [54]. Measurements of mitochondrial dGTP levels from fibroblasts of affected patients demonstrate that the reduction in dGTP correlates with the loss of mitochondrial copy number [55]. These studies thus support the notion that although dGTP is made through both *de novo* and salvage pathways, the salvage pathway is necessary to maintain mitochondrial DNA copy number.

Several diseases result from defects in enzymatic steps in thymidine salvage, including thymidine kinase II and thymidine phosphorylase [32]. Thymidine kinase II is critical for mitochondrial maintenance but the deficiency is myopathic and patients exhibit severe skeletal muscle weakness; infantile death generally results from respiratory failure [56,57]. However, substitutions at less-conserved amino acids, T230A and R225W, results in PEO, which is late in onset [56].

Thymidine phosphorylase deficiency is the cause of MNGIE [9,58,59], an extremely rare autosomal recessive disease. The clinical symptoms include gastrointestinal dysmotility, cachexia, ophthalmoparesis, and peripheral neuropathy [9,58]. Onset is generally within the first twenty years, although late-onset cases have also been reported. There is generally great variability in the pathology of the disease, even among siblings [59]. Missense mutations in thymidine phosphorylase correlate with late-onset MNGIE. Due to the onset of the disease in young adults, it can be misdiagnosed as anorexia. Onset of death often occurs in middle age (30–40 years) [9,58,59].

Interestingly, plasma deoxythymidine levels are elevated in MNGIE patients, while cellular mitochondrial DNA is depleted [60]. The progression of the disease correlates with mitochondrial DNA instability; however, elevated deoxypyrimidine levels may not correlate with affected tissues [60]. Although it is unclear why elevated levels of deoxythymidine would correlate with mitochondrial instability, recent reports suggest that mitochondrial concentrations of dCTP levels are depleted. To date, there is no proven treatment for the disease [61]. Dialysis can lessen the clinical burden of toxic nucleosides. Recently a number of clinics have proposed to restore thymidine phosphorylase activity through cell transplantation using allogeneic hematopoetic stem cells to restore thymidine phosphorylase activity [62].

As well as being conferred by defects in TK II, PEO can also result from mutations in RRM2B; RRM2B is the gene that encodes the subunit of ribonucleotide reductase, p53R2, which is associated with ribonucleotide reductase in non-cycling cells. Mutations that delete p53R2 lead to early onset fatal depletion of mitochondrial DNA [63,64,65]. However, compound p53R2 alleles can confer MNGIE [64,65]. A dominant allele RRM2B was observed to confer an adult-onset PEO with multiple mitochondrial lesions [66]. These observations indicate that RRM2B is essential for mitochondrial DNA maintenance, but, depending on the allele, late onset disease may result.

### 3.3. Ataxia Telangietasia (A-T)

A-T is an autosomal recessive disease that has a broad spectrum of disease phenotypes; the incidence is 1/40,000 in the United States [12,13]. These include hypersensitivity to X-rays, cerebellar neuronal degeneration, cancer susceptibility, and immunodeficiency. A-T patients may also have complicated secondary clinical presentations [11,12]. The cellular phenotypes of A-T are complex: besides high frequencies of chromosomal abnormalities, there are also mitochondrial dysfunctions and cytoskeletal abnormalities [11,12]. These complex cellular phenotypes may contribute to the neurodegeneration and ataxia that afflicts A-T patients.

The ATM kinase is a dimer that phosphorylates a large number of substrates and regulates cell cycle progression at G2. The ATM kinase not only is found in the nucleus, but also in the cytoplasm in differentiated non-proliferating neurons and in Purkinje cells. Disruption of ATM signaling leads to disruption of ribonucleotide reductase’s ability to upregulate mitochondrial DNA synthesis after ionizing radiation exposure, and early passage of A-T fibroblasts show 50% lower levels of mitochondria, perhaps due to faulty regulation of ribonucleotide reductase [67]. Eaton *et al.* [67] suggest that ATM-induced regulation of ribonucleotide reductase is essential for mitochondrial biogenesis. ATM also contributes to maintaining dNTP levels by nucleotide salvage. ATM can phosphorylate deoxycytidine kinase, thereby shifting the kinase substrate specificity towards deoxycytidine to maintain dNTP pools [10]. Thus, A-T could also contribute to mitochondrial stability through upregulating ribonucleotide reductase and maintaining dNTP balance when cells are exposed to agents that cause double-strand breaks.

## 4. Animal Models

Animal models are useful in untangling the complexities of disease phenotypes that are conferred by human metabolic syndromes in nucleotide metabolisms (see Table 2, for summary), as well as testing for potential therapeutic interventions that could restore deoxynucleotide balance and mitochondria [44]. Such complexities include the tissue specificities of genetic defects and disease onset and penetrance. Since biochemical pathways and genes in nucleotide metabolism are essentially conserved between rodents and man, it is now possible to create humanized mice that express particular disease-associated alleles of nucleotide biosynthetic genes.

### 4.1. Mouse Models for Lesch-Nyhan Syndrome

#### *Hprt^−/−^* Mice

Targeted deletion of *Hprt* in mice (*Hprt*^−/−^) does not give rise to any behavioral phenotypes suggestive of the characteristics of Lesch-Nyhan syndrome, namely mental retardation and the pathological self-mutilation [68,69]. Furthermore, despite having no detectable HPRT enzyme activity in the brain, these mice have normal brain purine content, an effect that was attributed to an increase in *de novo* purine synthesis [69], which also renders the mice hyperuricemic. Abnormal purine content has been noted for specific cell types, such as the astrocytes [70,71], and mutant mice exhibit age-related decrease in dopamine content in the brain [71]. It is important to note that other pathogenic human variants of HPRT have been documented that are associated with a less severe manifestation of Lesch-Nyhan syndrome; therefore, in any animal model of LNS, residual HPRT enzyme activity should be an important consideration when interpreting phenotypes [72].

### 4.2. Mouse Models for MDS Linked to Thymidine Kinase Deficiency

#### *Tk2^−/−^* Mice

Homozygous deletion of *Tk2* in mice (*Tk2^−/−^*) results in complete postnatal lethality between 2–4 weeks of life with multiple organ systems affected, likely due to organism-wide cellular mitochondrial defects including decreased mitochondrial DNA and abnormal crista morphology [73]. Another Tk2 targeted mouse carrying an amino acid substitution (*Tk2^H126N^*) found in a pediatric patient with MDS may represent a better model for this disease; similar to the *Tk2^−/−^* mouse, it displays complete postnatal lethality and mitochondrial defects, but there are also clear manifestations of neurological malfunction including tremors, weakness, decreased activity and altered gait [74].

Point mutations or deletions in mitochondrial DNA are not observed in *Tk2^−/−^* mice, perhaps due to the depletion of intracellular thymidine and subsequent cessation of mitochondrial DNA replication [75]. By day fourteen, mitochondrial DNA is depleted in skeletal muscle, heart, liver, adipose and spleen, thus affecting both resting and replicating tissue [75]. There is active investigation into whether the TK2 defect can be rescued by gene therapy; over-expression of TK2 in the mouse models extends the life span of *Tk2^−/−^* mice from three weeks to twenty months, suggesting that some clinical features of the disease might be reversible [76].

**Table 2:** Mouse models for human genetic defects in nucleotide salvage and metabolism.

Disease	Gene	Mouse Genotype	Phenotypes		
			Neurological	DNA Damage Sensitivity	Other
Lesch-Nyhan	*HGPRT1*	*Hprt^−/−^*	None reported	None reported	Hyperuricemia
MDS	*TK2*	*Tk2^−/−^*	None reported	None reported	Post-natal mortality (2–4 weeks); growth retardation; cellular mitochondrial defects; hypothermia due to the absence of subcutaneous adipose tissue; abnormal morphologies of brown adipocytes, myocardiocytes and hepatocytes
MDS	*TK2*	*TK^H126N^* (knock-in)	Encelphalopathy; tremors; weakness; decreased activity; altered gait	None reported	Post-natal mortality with defects similar to that of *Tk2^−/−^* mice
MNGIE	*TYMP* (*ECGF1*)	*TP^−/−^*; *UP^−/−^*	Encephalopathy; abnormal myelin sheath morphology; mitochondrial DNA instability in the brain	None reported	Elevated plasma thymidine; defects in nucleotide homeostasis and enzyme/coenzyme metabolism
MDS	*RRM2B*	*Rrm2b^−/−^*	Abnormal sciatic nerve morphology	Higher rates of spontaneous mutation in the kidney	Renal organ failure at 14 weeks
Ataxia telangiectasia	*ATM*	*Atm^−/−^*	Abnormal neuronal cell morphologies; neuronal cell degeneration	Hypersensitivity to gamma radiation; abnormal cell cycle checkpoint response; spontaneous lymphomas	Growth retardation; premature death; decreased thymocyte numbers; infertility in both sexes

TK2 is thymidine kinase 2; TYMP is thymidine phosphorlase; RRM2B is the gene encoding p53R2, a small subunit of ribonucleotide reductase that binds p53; ATM is the human gene “Mutated in ataxia telangiectasia”; MNGIE refers to mitochondrial neurogastrointestinal encephalomyopathy; TP is the gene encoding thymidine phosphorylase; UP is the gene encoding uridine phosphorylase.

### 4.3. Mouse Models for Mitochondrial Neurogastrointestinal Encephalomyopathy (MNGIE)

#### *TP^−/−^* and *UP^−/−^* Mice

Targeted deletion of thymidine phosphorylase (TP) in mice was hypothesized to give rise to an increase in plasma levels of thymidine and the disease pathologies associated with mitochondrial neurogastrointestinal encephalomyopathy (MNGIE). This hypothesis was not completely born out since the *TP^−/−^* mice have only modest increases in plasma thymidine and TP activity is maintained in the small intestine, likely due the compensatory action of uridine phosphorylase, which in mice has the ability to cleave both thymidine and uridine [76]; therefore, Lopez *et al.* [76] generated a TP and uridine phosphorylase UP double knock-out mouse (*TP^−/−^*; *UP^−/−^*), which did indeed increase the plasma levels of thymidine over those observed in the *TP^−/−^* single knockout mouse. Furthermore, *TP^−/−^*; *UP^−/−^* exhibit similar pathologies as human MNGIE patients.

### 4.4. Mouse Models for p53R2 Deficiency and Over-Expression of Ribonucleotide Reductase (RNR) Subunits

#### *Rrm2b^−/−^* Mice

The supply of cellular deoxyribonucleotides (dNTPs) pools is generated through the activity of multi-subunit ribonucleotide reductase enzymes, and p53R2 is a small subunit of this complex. To determine the role of this individual subunit in dNTP supply, Kimura *et al.* [77] generated a homozygous knock out mouse for Rrm2b (*Rrm2b^−/−^*) and found that the mice develop normally until they are weaned, after which they become severally growth retarded and die prematurely. Pathological features associated with this early mortality phenotype included increased DNA damage in the kidney and severe renal failure by the age of 14 weeks, suggesting that p53R2 is required for maintaining DNA-repair related dNTP pools in some organs [64]. Another intriguing link between p53R2, dNTP pools and the mitochondria emerged from a study that identified several mutations of the RRM2B in unrelated individuals with MDS, suggesting that p53R2 activity is also important for mtDNA replication [64].

Over-expression of p53R2 by gene therapy has been suggested as a means to increase levels of dNTPs in non-proliferating cells and restore mitochondria in *Rrm2b^−/−^* mice. Over-expression of RNR subunits, Rrm1, Rrm2, and p53R2, in transgenic mice, however, does not increase mitochondrial copy number, but instead leads to depletion of mitochondria [78]. Yikallio *et al.* [78] hypothesize that depletion of mitochondria in transgenic mice results from dNTP imbalance and show that ratios of dNTPs in mouse skeletal muscle are imbalanced, compared to wild type. These studies show that although p53R2 is necessary for maintaining dNTP levels, it is not sufficient.

### 4.5. A-T Mouse Models and Neurodegeneration

A-T mouse knockouts have been intensely studied. A-T mutant mice mimic human conditions, namely radiosensitivity, immunodeficiency, and cancer predisposition [79]. Although A-T mice do exhibit neurological abnormalities and have behavioral phenotypes, they do not exhibit progressive neurodegeneration. Cells from A-T mice appear to be under constant stress and exhibit high levels of the transcription factor activator protein-1 (AP-l) [80]. These studies indicate that although it is possible to mimic the DNA repair defect, it is not yet possible to mimic the neurodegenerative defect in the A-T mouse model [80].

## 5. Summary and Future Directions

DNA repair and nucleotide salvage mechanisms play an important role in preventing neurodegenerative disease. The purpose of this review was to consider how dNTP balance is maintained in nervous tissues, and how lack of salvage pathways leads to neurodegenerative diseases, resulting from DNA mutation. The primary conclusion is that nucleotide imbalance can lead to a wide variety of neurodegenerative diseases resulting from multiple changes in neural physiology. MDS diseases and associated neurological syndromes show clear correlation with deficiencies in mitochondrial dNTP levels, which lead to neurodegeneration resulting from defective mitochondrial DNA replication. On the other hand, LNS diseases, although linked to a single defect in nucleotide salvage, have complicated symptoms in which DNA repair has only recently been associated with metabolism.

Of the diseases discussed, both LNS and the hepatocerebral form of MDS resulting from mutations in dGUOK have the most severe neurological symptoms. Both involve salvage of guanine and illustrate the importance of guanine nucleotides in maintaining the physiology and the genetic integrity of the nervous system. Recent studies have suggested that HPRT1 functions in multiple housekeeping roles, including cell cycle and DNA repair [44]. It is important to note that neurological presentations are manifested in patients that have the most severe HPRT deficiencies, although further study is necessary to match genotype with disease progression [34].

MNGIE patients exhibit a wide variety of symptoms; the disease is later in onset than many MDS patients afflicted with the hepatocerebral form. Since the fidelity of mitochondrial replication is severely compromised when the mitochondrial nucleotide pool is insufficient or imbalanced, it is likely that defects resulting in the most imbalanced nucleotide pools confer an early and severe disease onset. Factors that account for the variable disease manifestations of MNGIE have not been fully elucidated. While the pathology was first thought to result from excess levels of deoxythymidine and deoxyuridine [60], it is now apparent that other nucleotides, such as dCTP, are actually deficient and may contribute to neurodegeneration [61]. These studies illustrate that understanding the broad spectrum of nucleotide imbalance may be necessary in unraveling the complexities of MNGIE.

Animal rodent models have limitations when studying late or gradual onset neurological degeneration but do show promise in studying genetic alleles. One interesting similarity between humans and mice is that many of the defects in nucleotide salvage do not present a phenotype until after weaning; this suggests that an alternative source of dNTPs may be available, such as a maternal source. However animal models also have limitations in understanding disease phenotypes. First, life span is shorter, so neurological defects that take years to develop in humans may not be manifested. Second, mice have higher levels of plasma thymidine and uridine, so the incremental increase in dNTPs resulting from genetic defects may have different physiological results.

The apparent “cure” for the diseases is to “correct” the genetic defects by increasing the insufficient nucleotide levels and restoring nucleotide pools. Indeed, increasing nucleotide pools appears sufficient to minimize genetic instability phenotypes in budding yeast mutants with defective deoxynucleotide levels [81]. Although increasing overall levels of deoxynucleotides appears sufficient to increase mitochondrial genomes in budding yeast [82], similar experiments in transgenic mice have not yielded the same results [64]. Some attempts to alleviate neurological symptoms of LNS patients by administration of *S*-adenosyl methione resulted in one published report of a positive outcome [83]. However, with the advent of stem cell technologies, new efforts are focusing on whether enzymatic deficiencies can be restored, using emerging transplant therapies [84].
